# Quantitative home-based assessment of Parkinson’s symptoms: The SENSE-PARK feasibility and usability study

**DOI:** 10.1186/s12883-015-0343-z

**Published:** 2015-06-10

**Authors:** Joaquim J. Ferreira, Catarina Godinho, Ana T. Santos, Josefa Domingos, Daisy Abreu, Raquel Lobo, Nilza Gonçalves, Marcio Barra, Frank Larsen, Øyvind Fagerbakke, Ingvild Akeren, Hilde Wangen, J. Artur Serrano, Peter Weber, Andrea Thoms, Stefan Meckler, Stefan Sollinger, Janet van Uem, Markus A. Hobert, Katrin S. Maier, Helen Matthew, Tom Isaacs, Joy Duffen, Holm Graessner, Walter Maetzler

**Affiliations:** Clinical Pharmacology Unit, Instituto de Medicina Molecular, Lisbon, Portugal; Laboratory of Clinical Pharmacology and Therapeutics, Faculty of Medicine, University of Lisbon, Lisbon, Portugal; CNS – Campus Neurológico Sénior, Torres Vedras, Portugal; Center for Interdisciplinary Research Egas Moniz (CiiEM), Cooperativa de Ensino Superior Egas Moniz, Monte de Caparica, Caparica, Portugal; NST-Norwegian Centre for Integrated Care and Telemedicine, University Hospital North Norway, Tromsø, Norway; Department of Clinical Medicine, Faculty of Health Sciences, The Arctic University of Norway, Tromsø, Norway; Hasomed GmbH, Magdeburg, Germany; HSG-IMIT, Villingen-Schwenningen, Germany; AbilityNet, London, UK; Hertie Institute for Clinical Brain Research, Department of Neurodegeneration, Center of Neurology, University of Tuebingen, Tuebingen, Germany; DZNE, German Center for Neurodegenerative Diseases, Tübingen, Germany; The Cure Parkinson’s Trust, London, UK; Institute for Medical Genetics and Applied Genomics, University of Tübingen, Tuebingen, Germany

**Keywords:** Parkinson’s disease, Self-assessment, Home-based, Usability, Feasibility

## Abstract

**Background:**

Currently, assessment of symptoms associated with Parkinson’s disease is mainly performed in the clinic. However, these assessments have limitations because they provide only a snapshot of the condition.

**Methods:**

The feasibility and usability of an objective, continuous and relatively unobtrusive system (SENSE-PARK System), which consists of wearable sensors (three worn during the day and one worn at night), a smartphone-based App, a balance board and computer software, was tested 24/7 over 12 weeks in a study including 22 PD patients. During the first four weeks of the study, patients did not get feedback about their performance, during the last eight weeks they did. The study included seven clinical visits with standardized interviews, and regular phone contact. The primary outcome was the number of drop-outs during the study. As secondary outcomes, the Post-Study System Usability Questionnaire (PSSUQ), score and information obtained from the standardized interviews were used to evaluate the usability of the system.

**Results:**

All patients completed the study. The participants rated the usability of the SENSE-PARK System with a mean score of 2.67 (±0.49) on the PSSUQ. The interviews revealed that most participants liked using the system and appreciated that it signaled changes in their health condition.

**Conclusions:**

This 12 week controlled study demonstrates that the acceptance level of PD patients using the SENSE-PARK System as a home-based 24/7 assessment is very good. Particular emphasis should be given to a user-friendly design. Motivation to wear such a system can be increased by providing direct feedback about the individual health condition.

## Background

At present, measurements of Parkinson’s disease symptoms are almost all performed in a clinical setting, which may not reflect daily life situations. The most widely used assessment scale for PD symptoms is the Unified Parkinson’s Disease Rating Scale (MDS - UPDRS) which includes interviews asking the patient for historical information referring to the previous week, and a clinical rating scale which includes semi-quantitative assessment of motor (dys) function [1]. However, there is broad agreement in the scientific field that new assessment strategies are needed, in particular those which have high ecological validity, multiple time points of evaluation and are effective [2].

Quantitative assessments using wearable technology may allow for continuous, objective and ecologically valid data collection and can be applied frequently at short intervals outside of the physician’s office, allowing real-time monitoring of symptom changes. This approach may also improve patient-doctor interaction, influence therapeutic decisions and ultimately ameliorate patients’ global health status. In addition, such measures have the potential to be used as outcome parameters in clinical trials, allowing for frequent assessments (e.g., in the home setting). These wearable sensors are of particular interest as they can be worn unobtrusively, so they do not relevantly influence the person who wears the sensor during a test, or during daily life. In addition, they can measure movements, and can be attached to almost every part of the body where symptoms of interest can occur [2].

The first studies with such sensors were performed more than 10 years ago and focused on the assessment of tremor and dyskinesia. Feeding the information obtained by such sensors back to the individual user, so that he or she can learn more about the individual disease presentations and how to counteract disease-associated symptoms, may provide additional motivation for users.

Within the framework of an EU-funded project (www-sense-park.eu), a device consisting of four components: software, a smartphone app, a Wii balance board and a set of sensors, was developed [3]. This paper focuses on the results of this study with regard to feasibility and usability of the SENSE-PARK System over a prolonged time frame in the home environment of the users.

## Methods

A multi-centre, open-label, feasibility and usability study comprising 22 participants was conducted. Eleven PD patients were invited to use the SENSE-PARK System for 12 weeks and perform clinical assessments every other week. The other 11 patients only completed the clinical assessments.

The primary outcome was the frequency of drop-outs and the first secondary outcome was assessed through the IBM Post-Study Usability Questionnaire (PSSUQ - rating score from 1, best, to 7, worst).

### Technology overview

**Fig. 1:**
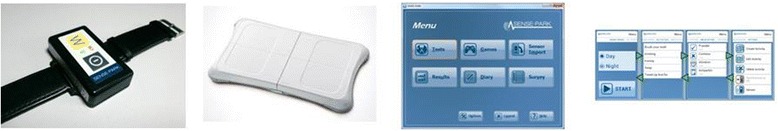
From left to right: Inertial sensor unit with housing and wristlet band; Wii balance board; interface software; SENSE-PARK App

The SENSE-PARK System consists of a set of wearable sensors (3 to be used during the day and one at night), a Wii Balance Board, software and a smartphone app (Fig. 1).

The sensors monitor movements of PD patients during daily activities, collecting motor-related raw data. Accelerometers and angular rate sensors measure the motion of the user, and map certain activities. When awake, the user wears a small sensor at the wrist and the leg of the more affected side, as well as at the lower back according to Fig. 1. When asleep, the user wears one sensor at the lower back only. This set of sensors, together with algorithms developed during the SENSE-PARK project phase, allows monitoring parameters associated with gait, hypokinesia, dyskinesia, tremor and sleep. Through the Wii Balance Board, data such as body weight and sway are also collected. The user also performs cognitive tests through specific software. These tests use virtual environments on a screen to evaluate specific cognitive domains, including alertness, divided attention, response control, visual exploration and working and topological memory.

### Staff Training

A two day investigator meeting was conducted to train staff, including installation of equipment, and procedures for training participants in the use of the System. Investigators received training in test administration and scoring.

### Participants

A sample of 22 PD patients was divided into two groups: SENSE-PARK System users and non-users. Inclusion criteria were (1) PD patients between 40 and 85 years of age, (2) stage 1 to 2.5 (ON) of the Modified Hoehn and Yahr (H&Y) scale [4] and (3) having experience or interest in technical equipment (computer, regular mouse and keyboard).

Exclusion criteria included (1) illiteracy, (2) ≤24 in the Mini Mental State Examination (MMSE) [5], (3) postural instability item MDS-UPDRS > 2, (4) inability to handle the device for some other reason.

There were no restrictions on prior and concomitant therapy. An approval was obtained from the local ethics committee at the three sites (University of Lisbon, Portugal; University of Tübingen, Germany; and University of Tromsö, Norway), and in compliance with national legislation and Declaration of Helsinki.

### Study design and assessments

After signing the informed consent, participants were screened for eligibility. For the group of users, a home visit was scheduled to ensure users had the required home facilities for the use of the SENSE-PARK System and to install the full system (the mouse setting was defined according to the hand the user normally writes with). The users were also invited to perform a demo session, in order to get acquainted with the SENSE-PARK System. Only balance and cognitive testing had to be performed “actively”. On cognitive test occasions, patients were instructed to be seated in a chair in front of the computer and place the computer and mouse on a table. Balance tests had to be performed with a Wii Balance Board which was connected to the local computer and the SENSE-PARK System. The other domains’ data were collected with the sensors which were worn 24 hours per day: three to be used during the day and one at night.

After the installation, participants used the SENSE-PARK System at home for one week and then they were observed by the investigator who registered whether the participants made mistakes during the tasks and whether they needed assistance. When necessary, another week of training was allowed. The time needed for confident SENSE-PARK System operation, test taking, data storage and data transfer was recorded. As soon as the user was able to use the SENSE-PARK System adequately and it was working properly, the assessment phase of the study started.

During the assessment period, patients who used the SENSE-PARK System were asked to perform sway assessment and cognitive tests every other day at a similar time of day during ON stage. Patients selected, in advance, the days of the week they would perform their testing, which was then programmed into the SENSE-PARK System with a reminder coming up on the interface of the software. An ongoing check of data arrival allowed users who were near to missing their two day window to be contacted by the study staff. If any problems or questions developed, users had access to the study staff for backup support.

Every other week during the study, participants from both groups returned to a health professional expert in movement disorders or were visited to perform the control of concomitant medications, check data download, record the occurrence of any Adverse Event, confirm patient compliance and to perform the following rating scales: MDS-UPDRS [1], H&Y [4] , Montreal Cognitive Assessment (MoCA) [6], Mini–Mental State Examination (MMSE) [5], Parkinson's Disease Questionnaire-39 (PDQ-39) [7], EQ-5D [8], Epworth Sleepiness Scale (ESS) [9], Panic Disorder Severity Scale (PDSS) [10], Non-Motor Symptoms Scale (NMSS) [11], Unified Dyskinesia Rating Scale (UdysRS) [12], Clinical Global Impression - Severity Scale and Improvement Scale (CGI-S and CGI-I) [13].

Between investigator visits, semi-structured interviews were conducted by phone to gain insight into the experiences of the participants using the SENSE-PARK System. Topics discussed were: willingness to continue in the study, satisfaction with the SENSE-PARK System, changes in health status or medical condition, adverse events, feedback messages and doubts about the system. Those participants who were not using the SENSE-PARK System were asked about willingness to continue in the study, health status, medical condition and adverse events (usability evaluation).

**Fig. 2:**
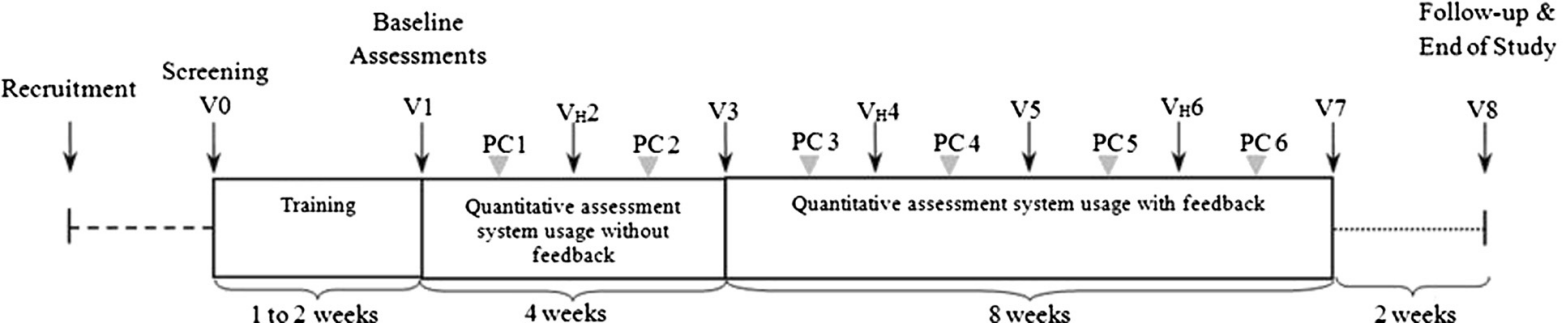
Study design. V: Visit; PC: Phone Contact; VH: Home visits

After 4 weeks of active data collection with the SENSE-PARK System, the participants received a modified version of the software. This new version provided feedback to users (Fig. 2).

At the end of the study period (V8), participants were asked to fill in the IBM Post-Study Usability Questionnaire (PSSUQ) [14] which assesses user satisfaction with, and usability of, technical devices. It is a 19-item closed-ended ordinal questionnaire, based on 7-point graphic Likert scales. The items address five important components of user satisfaction with general computer systems usability: ease of use, ease of learning, simplicity, effectiveness, information, and the user interface [15].

### Statistical analysis

Statistical analyses were performed in R, version 3.0.3. Subject demographic and clinical data were examined by descriptive summary. Usability was assessed for system users and was tested with the PSSUQ values obtained at the end of the study (see above). Feasibility was assessed for users and non-users. For non-users, feasibility was assessed through willingness to continue in the study and through completeness of the study, i.e. withdrawals from the study. For users, feasibility was assessed through the following indices: willingness to continue in the study and through completeness of the study, duration of time to train participants to use the SENSE-PARK System confidently, compliance in terms of the number of completed test sessions and their timeliness with every other day assessments, compliance/adherence to system measures, success of data transfer and decryption, ease of use and program satisfaction [16, 17].

## Results

Twenty two idiopathic PD patients (14 male, 8 female) were included in the study (Table 1). There were no significant differences regarding age and sex distribution as well as MMSE scores among the countries. Although not significantly different, study participants included in Norway were slightly younger.

**Table 1:** Baseline Characteristics

	Germany		Norway		Portugal		p-value
	User	Non-User	User	Non-User	User	Non-User	
Nr or participants (male)	5 (3)	5 (3)	3 (0)	3 (2)	3 (3)	3 (3)	0.09
Age (mean ± sd)	60.2 (9.8)	60.6 (10.5)	59.3 (3.7)	53 (9.9)	66 (2.7)	60 (6.2)	0.37
HY (median, range)	2 (2–3)	1.5 (1–1.5)	2 (2–2)	1 (1–2)	2 (1–2)	2 (2–2)	1.00
Total MDS-UPDRS (median, range)	61 (53–76)	61 (49–45)	52 (46–56)	26 (22–31)	50 (26–65)	60 (45–70)	0.23
MDS-UPDRS part III (median, range)	40 (28–42)	17 (11–29)	17 (16–24)	15 (10–15)	26 (15–37)	40 (38–44)	0.05
Total MMSE (median,range)	30 (29–30)	29 (29–30)	29 (29–30)	30 (29–30)	29 (29–30)	28 (26–30)	0.60

### Feasibility aspects

#### Users

**Fig. 3:**
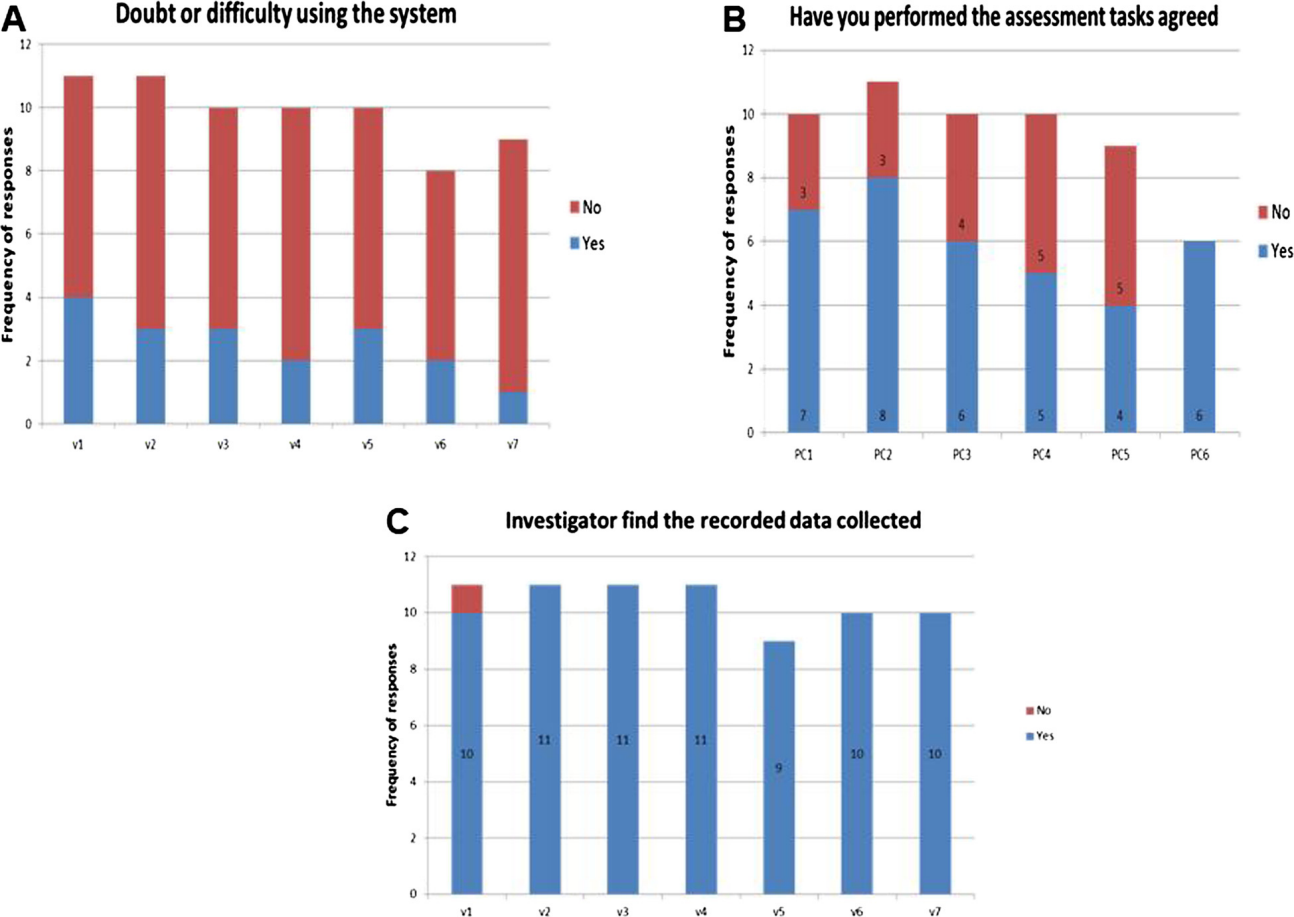
**a** Bar plot showing the frequency of doubts along the study; **b** Number of tasks preformed; **c** Number of times the investigator found the recorded data

All participants showed willingness to continue in the study from visit 1 to visit 8, and all of them completed the study. Most participants completed the tasks asked for during the study and according to the protocol. Number of doubts and difficulties decreased along the study (Fig. 3). Only one participant failed to transfer the data when asked. All the other SENSE-PARK System users were able to download the data along the study.

#### Non-users

All participants showed willingness to continue in the study from visit 1 to visit 8, and all of them completed the study. Since these patients were not system users questions concerning data transfer, number of doubts and difficulties and completion of tasks were not assessed.

No adverse events were recorded.

### Usability aspects

**Fig. 4:**
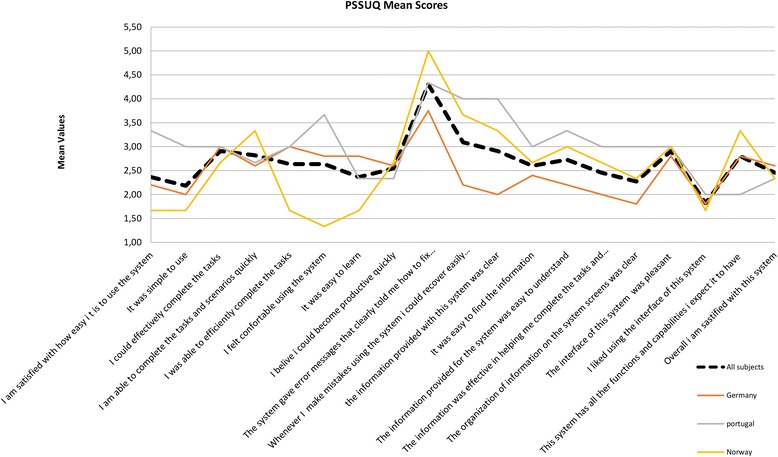
Plot showing the mean values of responses for the PSSUQ scale divided by country

Usability aspects were only assessed for system users. Overall analyses from the PSSUQ scale showed that the system was generally well accepted (Fig. 4). Analysis of the PSSUQ scale by country showed that study participants from Norway had a slightly different profile than those from the other countries, scoring less (i.e. better) with regard to system use in items such as ability to efficiently complete tasks, comfort with using the system, and ease of learning.

#### Problems that arise from using this device

There were four adverse events registered, three of them being classified as definitely related to the study and one being possibly related to the study (Table 2).

**Table 2:** Adverse events description

	Adverse Events		
Group	Number of cases	Country	Description
User	2 cases	Portugal	Allergy to plastic bracelet
			Loss of wrist sensor
	1 case	Norway	Minor stroke
	1 case	Germany	Broken sensor
Non-user	No adverse events were reported		

## Discussion

We found the SENSE-PARK System highly feasible in terms of patient compliance, satisfaction and ease of use. Patients maintained their involvement with the program over 16 weeks, and several requested a continuation of the program at study end. We were initially concerned that participants would find the testing process, of every other day participation and weekly visits, tedious, but the satisfaction ratings did not support this concern, and the computer-based technology guided the test-taking smoothly. However, we encountered more problems with transmission and sensor errors compared to other tasks, but by mid-study, technical correction was achieved.

Hardware related problems consisted mainly of a broken sensor. Battery replacements were not required.

The training period was short and the testing was self-administered without direct investigator involvement. Consistent with the concept of the SENSE-PARK System as a monitoring tool, after the first 4 weeks we allowed users to see their previous scores when they took their next test.

## Conclusions

This pilot study is a “proof of concept” and establishes patient and technical feasibility of the SENSE-PARK system.

There is, as yet, no evidence that using this test array will improve treatment of patients during clinical practice or that a cost benefit analysis is favorable. Further studies are needed to test how the test array performs in a clinical setting with larger patient populations. One question to be addressed is whether the SENSE-PARK System can detect a need for treatment changes. Additionally, whether there are country-specific issues, creating different requirements for its introduction into clinical practice, needs to be evaluated.

## Abbreviations

CGI-I: Clinical global impression - Improvement scale
CGI-S: Clinical global impression - Severity scale
EQ-5D: EuroQol-5D
ESS: Epworth sleepiness scale
H&Y: Hoehn and Yahr
MDS-UPDRS: (Movement disorder society) Unified Parkinson’s disease rating scale
MMSE: Mini–mental state examination
MoCA: Montreal cognitive assessment
NMSS: Non-motor symptoms scale PDQ-39, Parkinson's disease questionnaire-39
PDSS: Panic disorder severity scale
PSSUQ: Post-study system usability questionnaire
UdysRS: Unified Dyskinesia rating scale
